# Blood Transfusion Practice among Healthcare Personnel in Nepal: An Observational Study

**DOI:** 10.1155/2018/6190859

**Published:** 2018-02-12

**Authors:** Abja Sapkota, Sabitra Poudel, Arun Sedhain, Niru Khatiwada

**Affiliations:** ^1^Nepal Medical College, Department of Nursing, Kathmandu University, P.O. Box 13344, Fax No. 977-1-4912118, Jorpati, Kathmandu, Nepal; ^2^Kathmandu Medical College, Department of Nursing, Kathmandu University, P.O. Box 21266, Fax No. 977-1-4477920, Sinamangal, Kathmandu, Nepal; ^3^Chitwan Medical College, Department of Medicine, Tribhuvan University, P.O. Box 42, Fax No. 056-592364, Bharatpur, Chitwan, Nepal

## Abstract

**Background:**

The complications associated with errors in transfusion practice can be minimized by assessing transfusion practices. In Nepal, there is no standard protocol on blood transfusion. So, this study was conducted with an aim to assess the blood transfusion practice among healthcare personnel.

**Methods:**

A descriptive observational study was conducted in two tertiary hospitals in Kathmandu, Nepal, over a period of 10 months. Bedside blood transfusion procedures were observed using structured checklist.

**Results:**

Altogether, 86 observations were made. Time taken from dispatch from the blood bank to transfusion was >2 hours in 53.2% of cases. In majority of the cases, blood was kept in the ward in uncontrolled and unprotected manner by the patients' relatives. Only 8.2% of the patients and/or the relatives were informed about the reasons, associated probable risks (2.4%), and the benefits of transfusion (4.7%). Assessment of vital signs at 15 minutes of initiation of transfusion was done on about 2 to 4% of cases.

**Conclusion:**

We found a suboptimal blood transfusion practice in Nepal, which could be attributable to substantial knowledge gap among healthcare personnel and the absence of quality culture, quality system, and quality management in the area of blood transfusion practices.

## 1. Introduction

Blood transfusion has well proven clinical benefit in the treatment of anemia where it helps by improving oxygen delivery to tissues [1]. The trend of blood transfusion is increasing globally, which has risen from 85 million units of transfusion in 2012 [2] to 112.5 million of donations in 2016 [3]. According to World Health Organization global data base system (WHO-GDBS) report, 2016, a total of 255,178 blood products were issued and transfused in Nepal, of which 149,635 were whole blood and 51,487 were red blood cells (RBCs) [4].

Despite its beneficial effects, blood transfusion procedure might be risky at times with development of many adverse events. It may result in acute or delayed complications ranging from the development of acute hemolytic reaction, transfusion reaction, febrile reaction, and septic reaction. It carries the risk of transfusion-transmissible infections including HIV, hepatitis viruses, syphilis, malaria, and Chagas disease [5, 6].

A total of 3,288 cases were reported in the serious hazard of blood transfusion (SHOT) report in the year 2015. Of the total SHOT cases, 77.7% of errors resulted from mistakes or “human factors,” and only 10% were not preventable (mostly allergic/febrile reactions). The number of cases with major morbidity was 166 and the total deaths reported were 26. The reported major morbidities were hemolytic transfusion reactions, transfusion associated circulatory overload (TACO), and transfusion-transmitted infection, transfusion-related acute lung injury (TRALI), ABO incompatible transfusion, and transfusion of incorrect blood product. These complications could have occurred because of the errors in blood transfusion practice. Major contributing factors for these errors could be the problems in transportation of the blood constituent from blood bank to the hospital, lack of cross-checking practice at bed side, and lack of regular monitoring of the patients during and after the transfusion process [6–8].

There is a practice of standard guidelines or transfusion-related policies in different countries, which cover important processes of blood transfusion practice including the screening of donor blood for infectious diseases, analysis of the necessity of transfusion, and ABO compatibility tests [9–12]. Most of the practiced guidelines highlight the importance of proper identification of the patient that comprises complete name, date of birth, and hospital admission numbers given to each patient. These current guidelines further state that the patient undergoing blood transfusion should be assessed for not more than 60 minutes before transfusion and at 15 minutes of the commencement of transfusion [8, 13, 14].

In Nepal, there is a lack of proper blood transfusion policy and guidelines. Although the national guideline for blood transfusion was formulated in the year 2008, it mainly focused on selection of blood donors and blood donation criteria but lacked the specific protocol on safe blood administration procedures [15]. Probably because of the lack of proper guideline, healthcare providers do not follow the specific trends of keeping records of the transfusion-related adverse events. We do have very few studies done on blood transfusion in Nepal. One report prepared by WHO showed the lack of transfusion committee in all studied hospitals in Nepal [15]. So, this study was conducted to assess the existing blood transfusion practices among healthcare personnel in two teaching hospitals at central part of Nepal, which, we believe, would be helpful to provide baseline information in the process of preparation of a national guideline and protocol on blood transfusion procedure.

## 2. Methods

### 2.1. Study Design and Area

This was a descriptive observational study that was conducted over a period of 10 months from June 2016 to March 2017 at Nepal Medical College Teaching Hospital (NMCTH) and Kathmandu Medical College Teaching Hospital (KMCTH) in Kathmandu, Nepal. The studied hospitals had a number of similarities in terms of types of hospitals and bed numbers (both being teaching hospitals affiliated to the same university with 700 beds in each with a bed occupancy rate of 50–60%). Approval from the institutional review committee (IRC) of both hospitals was received beforehand.

A total of eighty-six blood transfusion procedures were observed in different wards of the hospital, namely, medical, gynecology and obstetrics, postoperative, surgical, high dependency unit (HDU), orthopedic ward, and the intensive care unit (ICU). The observation was made 30 minutes prior to initiation of blood administration till 20 minutes after the commencement of transfusion.

### 2.2. Data Collection Tools and Procedure

A structured observational checklist was prepared with reference to standard tools and guidelines used in previous studies and nursing standard of blood transfusion practices [9, 13, 14, 16], which was used to conduct the direct observation of the transfusion process of the healthcare personnel at the bed side. In few instances, direct interview with the patient's visitors and phone calls to the healthcare personnel involved in the transfusion was done to record the time of completion of transfusion. Utmost precaution was taken to reduce the possible biasness during the study. Written informed consent was taken from respondents before they were observed.

Faculties of medical and surgical nursing of the studied hospitals were consulted before finalizing the tool. Pretesting was done among eight healthcare personnel who carried out blood transfusion in the wards to assess the feasibility and practicability of the tools.

During the data collection period, enquiry was done in respective wards of the hospitals every day between 9 and 11 a.m. to find out the plans for blood transfusion on that day. If there were plans and prescription for blood transfusion, after proper arrangement of the blood from the blood bank, the researcher would reach the ward with standard tool at least 30 minutes before the initiation of transfusion.

The checklist consisted of three sections. Section A is comprised of information collected through records and observations, which included particulars of the patients including patient identification number, date and location of transfusion, details of the blood including blood bag number, ABO and Rh typing, blood collection date, and time of initiation of transfusion. Section B included observation of bedside blood administration procedure that included the checklist before, during, and after procedures. Section C consisted of details related to posttransfusion records.

### 2.3. Data Processing and Analysis

Checklist was checked every day for errors and the completeness. Data were entered and analyzed using the statistical package for social sciences V 16 (SPSS Inc., Chicago, IL, USA).

## 3. Results

A total of eighty-six blood transfusion procedures were observed. During the study period, the highest numbers of transfusions were carried out in medical and gynecology wards (27.9% each) followed by high dependency unit (11.6%), surgery (10.5%), and orthopedic wards (10.5%) (Table 1). Blood transfusion done at the hemodialysis (HD) unit was excluded from the study as the process and protocol followed in this unit are different from other wards.

**Figure 1:**
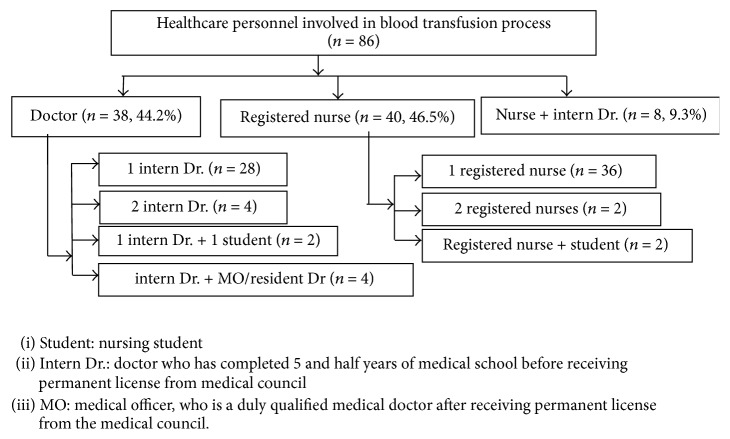
Healthcare personnel performing blood transfusion.

Figure 1 illustrates the personnel involved in blood transfusion process. Nurses were the key persons involved in the transfusion process followed by intern doctors with a percentage of 46.5 and 44.2, respectively. Both nurses and doctors were mutually involved in 9% of the transfusion events.

There was no proper practice of recording the details related to the time of dispatch of blood from the laboratory and completion of blood transfusion. So, the details regarding this information could not be traced in all but only in 77 observed cases, through direct contact with the caregiver who brought the blood to the patient's bedside. The median time taken from the dispatch of blood from the hospital blood bank/laboratory to initiation of transfusion was 2 hours and 15 minutes with a minimum time of 15 minutes to maximum time of 6 hours and 40 minutes. Similarly, the record of completion time of blood transfusion could be found out only in 62 observed cases through phone calls to the respective wards. The median time taken for transfusion of blood was 4 hours and 30 minutes that ranged from 1 hour to 6 hours and 55 minutes (Table 2).

Observations were also made whether the healthcare providers followed the standard blood transfusion guidelines and protocol during the procedure. Out of 86 observations, one blood transfusion was cancelled due to mismatch in the blood bag number mentioned in the cross-match sheet and blood unit itself. So, it was excluded from the study and only 85 cases were included for analysis. Only 8.2% of the patients and/or the relatives were informed about the reasons, associated probable risks (2.4%), and the benefits of transfusion (4.7%). Risks of transfusion that were expected to be explained to the patients and/or relatives were blood-borne infection, hemolytic allergic reactions, and febrile reactions [17, 18]. The benefits of transfusion to be explained were replacement of lost/deficient blood, treatment of weakness, improving oxygenation, and hence improving overall sense of well-being [19, 20] (Table 3).

The blood was first brought from the central blood bank in the cold box to the hospital blood bank by the patients' relatives, which was then kept in the refrigerator in controlled temperature. There was no trend of provision of cold box to bring blood from the laboratory to the wards. None of the hospitals provided clean hospital linen to warm up the blood.

During the preparation of blood transfusion, only 43.5% of healthcare personnel wore gloves during the procedure. The confirmation of the patency of the intravenous cannula was considered if the flushing was done with 0.9% normal saline solution before transfusion. Only 16.5% of personnel confirmed the patency of the intravenous (IV) cannula before initiation of the transfusion. Documentation of different product of transfusion like blood bag number, types of blood product, and blood volume was maintained in about 95% of cases. There were seven patients in bed side monitoring device.

The expected monitoring of vital signs was considered if done manually. Pretransfusion assessment of the patients was maintained in about 60% of patients. However, the percentage of recording of clinical parameters was higher during the transfusion procedure that ranged from 76.5 to 83.5% probably based on retrospective assumption (Table 4).

Out of the total 85 transfusions, one more was excluded again because the blood was clotted soon after the initiation of transfusion. So only 84 were included for the observation of practice after initiating transfusion. Time of initiation of transfusion was documented in 82.1% of cases and 21.4% of healthcare personnel stayed at the site of transfusion during 10–15 minutes of initiation of the transfusion. Recording of vital clinical parameters at 15 minutes of initiation of blood transfusion was very low that ranged from 2.4 to 4.8%. However, the interesting part of our observation was that despite low percentage of direct monitoring, records were maintained in slightly higher percentage of cases (ranging from 3.6 to 4.8%) (Table 5).

## 4. Discussion

This was the first study ever done in Nepal to analyze the blood transfusion practice among healthcare personnel. There was variation in the involvement of healthcare personnel for blood transfusion; out of total transfusions, seventy percent were carried out by single health personnel. The guidelines by WHO and British Committee for standards in hematology (BCSH) demand the involvement of at least one registered healthcare personnel who might be a doctor and or a nurse [9, 14]. These guidelines emphasize the necessity of verification of transfusion procedure by two people, one of whom is required to be registered healthcare personnel. Our finding in terms of spectrum of involved man power is similar to the observational study from Uganda [21]. These variations, especially seen in the developing countries, might be because of the lack of clear guidelines and unavailability of trained healthcare personal as well as the lack of uniformity in the institutional policy.

Transportation of blood units in open air, over long period of time and distances, may cause warming of the blood unit, which may result in bacterial growth and hemolysis of the blood [22, 23]. Almost all published guidelines suggest that the transfusion of blood should be started within 30 minutes and be completed within four hours after it is taken out of the controlled temperature of the refrigerator [24]. We found lack of practice in keeping record of time when blood was taken out of controlled temperature in the blood bank. Of the limited information that could be retrieved, very few numbers of transfusions were completed within four hours. This observation is similar to the report given by WHO in collaboration with other organizations in different centers of Nepal [15]. This finding of our study is similar to the blood transfusion practice in Uganda where blood was kept in warm temperature for about three hours before transfusion. However, In United Arab Emirates (UAE) more than fifty percent of transfusions were initiated within 30 minutes and completed within four hours after taking the blood out from the controlled temperature [16]. The delay in initiation and completion of the transfusion within the recommended time might be because of the lack of sufficient healthcare personnel and guidelines as well as the monitoring system in the hospital where blood transfusion took place.

In this study, blood was collected by the patients' caregiver from the hospital blood bank/ laboratory and brought to the hospital wards in their bare hands, which was later warmed by keeping it under armpits and sometimes by putting it inside the patient's blanket. Similar practice has been reported by de Graaf et al. [21]. This may be due to the lack of knowledge that blood should not be warmed unless and until fast transfusion at a rate of more than 100 mL per minute is needed [10, 13] and the lack of proper blood transfusion policy and clear guidelines and protocols in the hospitals. Most importantly, the absence of quality culture, quality system, and quality management in the studied centers might be the root causes for poor practices in blood transfusion, for example, having blood products transported in uncontrolled and unprotected manner by the patients' relatives/friends. Also, the substantial knowledge gap of healthcare personnel regarding bedside transfusion medicine and practices, for example, urging relatives to warm the blood product before transfusion by keeping it in their armpit/linen, might be another important contributing factor.

Only a few numbers of healthcare personnel (<10%), in our study, did not ask for cross-match sheet before initiating transfusion, without which the compatibility result and patients' identification would not have been found out. This practice could be labelled as the act of negligence on part of the healthcare personnel providing blood transfusion, which could, at times, have created catastrophic effects. Similar finding has been reported by Hijji et al. [16]. Error related to transfusion of incorrect blood component to the patients might be multifactorial [25] and it remains one of the largest risks related to transfusion. Nurses can increase compliance in high-risk areas of the transfusion process and reduce the probability of errors by developing accessible blood transfusion policies, auditable performance standards and training, and educational initiatives [26].

Less than half of the percentage wore gloves before initiating blood transfusion, which is lesser than in other studies [16]. The patency of the intravenous cannula before initiation of transfusion was checked by about sixteen percent of the cases. Generally, most of the healthcare providers use the commercial blood transfusion set available in the market with intravenous cannula of size 18 to 20 gauges.

Almost all cases of transfusion were started at a rate less than or equal to 2 ml per minute, which was in accordance with the recommended guidelines. Almost eighty percent mentioned starting time. About one-fifth stayed with the patient in initial 15 minutes. This is slightly less than another study where one-third stayed with patients in initial 15 minutes [16]. The duration of transfusion in our study was equal to or less than 4 hours in 33.9% of cases. The relatively inadequate duration of transfusion was probably due to the lack of knowledge about procedures or due to inadequate duration of transfusion per se. In one of the studies by Reis et al., duration of transfusion was inadequate in 8% of transfusions [27]. The frequency of monitoring and recording of vital signs in the initial period of initiation of transfusion was lesser in our study in comparison to another similar study [16]. However, our findings are similar to the study done in acute NHS trust in London, where observations made at fifteen minutes were only eighteen percent [28].

## 5. Strength and Limitation

The strong part of our study was that it was based on observation of bedside blood transfusion practice. Such type of study has been considered as an excellent method to assess the clinical performance of healthcare personnel. The weak part of it was inclusion of limited centers for the study. Only two private hospitals could be included in the study so the findings might not be generalized to other public hospitals, where the scenario might be further worse. As the participants knew beforehand that they were being observed, this could have caused bias in their actual practice and performance of the blood transfusion process.

## 6. Conclusion

This study showed that bed side blood administration practice was suboptimal in the context of Nepal. There was shortcoming in the quality culture, quality system, and quality management in terms of blood transfusion procedures and practices in the hospitals of Nepal. The results showed that there was substantial knowledge gap in healthcare personnel regarding clinical transfusion medicine and practice, which requires timely improvement in the formulation, implementation, and monitoring of blood transfusion policy and strategy, including appropriate guidelines. We would also like to emphasize the urgent need of orientation and training to the healthcare personnel on blood transfusion practices.

## Figures and Tables

**Table 1:** Hospital wards studied for blood transfusion practice (*N* = 86).

Areas of observation	Frequency	Percentage
Gynecology and obstetrics	24	27.9
General medicine	24	27.9
HDU	10	11.6
General surgery	9	10.5
Orthopedics	9	10.5
ICU	4	4.7
Others	6	6.9

HDU: high dependency unit; ICU: intensive care unit; and others: oncology (2), postoperative (3), and ENT ward (1).

**Table 2:** Time spent from dispatch of blood to transfusion (*N* = 77).

Duration	Frequency	Percentage
*Between dispatch from laboratory and transfusion *		
Less than 1 hr	8	10.4
1-2 hours	28	36.4
More than 2 hours	41	53.2
Median duration	2 hours 15 min	
Minimum	15 min	
Maximum	6 hours 40 min	
*Transfusion duration *(*n* = 62)		
Up to 4 hours	21	33.9
More than 4 hrs to 6 hrs	33	53.2
Above 6 hours	8	12.9
Median duration	4 hours 30 min	
Minimum	1 hour	
Maximum	6 hours 55 min	

**Table 3:** Practice of proper counselling before initiation of blood transfusion (*N* = 85).

Counselling before transfusion	Yes (*n*)	%
Explained patient and visitors about		
Reasons for transfusion	7	8.2
Risk of blood transfusion	2	2.4
Benefits of blood transfusion	4	4.7
Documentation regarding explanation on		
Reasons for transfusion	3	3.5
Risk of blood transfusion	2	2.4
Benefits of blood transfusion	1	1.2

Reasons for transfusion: blood loss, low hemoglobin, and anemia.

**Table 4:** Practice of proper techniques during preparation of blood transfusion (*N* = 85).

Preparation before blood transfusion	Yes	%
Used special box or tray to bring blood from lab	-	-
Blood warmed in clean hospital linen	-	-
Asked patient/visitors for cross-match sheet (*n* = 86)	79	91.9
Wore gloves	37	43.5
Confirmed patency of intravenous cannula	14	16.5
Asked patient to state full name	7	8.1
Documentation of		
Patients name that matched the cross-match results and other record sheets	74	87
Blood bag number (*n* = 86)	81	94.2
Blood collection date	81	95.2
Blood component expiry date	81	95.2
Blood group	81	95.2
Blood component	81	95.2
Blood volume	81	95.2
Preformed pretransfusion assessment within 30 min prior to blood transfusion		
Measured baseline blood pressure	51	60.0
Measured baseline pulse	53	62.3
Measured baseline temperature	52	61.1
Measured baseline respiration	47	55.0
Recorded pretransfusion vital signs in record sheet		
Recorded pretransfusion blood pressure	71	83.5
Recorded pretransfusion pulse	66	77.6
Recorded pretransfusion temperature	74	87.1
Recorded pretransfusion respiration	65	76.5
Used appropriate blood administration set	83	97.6

Appropriate set: transfusion set available in the market, cannula size of 18–20-gauge intravenous cannula; measurement of vital signs: considered measured if done by manual methods; confirmation of patency of cannula: considered if flushed with compatible solution before blood transfusion.

**Table 5:** Practice of monitoring after initiation of blood transfusion (*N* = 84).

After initiating transfusion	*N*	%
Transfusion started at rate ≤ 2 ml per min	84	100
Documented starting time	69	82.1
Stay for initial 10–15 min	18	21.4
Monitored and recorded at 15 min		
Blood pressure	2	2.4
Pulse	2	2.4
Respiration	2	2.4
Temperature	4	4.8
Records at 15 min		
BP	3	3.6
Pulse	3	3.6
Respiration	2	2.4
Temperature	4	4.8
Advice patient/visitor to report any unwanted symptoms if occurred to duty staff	76	90.5

Unwanted symptoms: allergies, itching, flushing, fever, and back pain.
